# Music intervention for reducing anxiety in patients undergoing CT or MRI examinations: a systematic review and meta-analysis of randomized controlled trials

**DOI:** 10.3389/fpsyt.2026.1864214

**Published:** 2026-08-05

**Authors:** Peng Hu, Lei Zhang, Jie Ren, Meng Wang

**Affiliations:** 1 Department of Medical Imaging, The People’s Hospital of Huantai, Zibo, Shandong, China; 2 Department of Pediatrics, The People’s Hospital of Huantai, Zibo, Shandong, China

**Keywords:** anxiety, computed tomography, magnetic resonance imaging, meta-analysis, music intervention

## Abstract

**Background:**

Computed tomography (CT) and magnetic resonance imaging (MRI) are essential diagnostic tools but often provoke significant anxiety in patients, which may compromise image quality and patient cooperation. Non-pharmacological interventions such as music have been proposed as feasible alternatives to anxiolytic medications. However, the evidence supporting music interventions in radiology settings has not been systematically synthesized.

**Objective:**

This meta-analysis aimed to evaluate the effect of music intervention on anxiety in patients undergoing CT or MRI.

**Methods:**

A systematic search was conducted in PubMed, EMBASE, Cochrane Library, Web of Science, and CINAHL from inception to March 1, 2026. Randomized controlled trials (RCTs) evaluating music interventions versus control conditions (e.g., usual care or headphones only) in patients undergoing CT or MRI were included. Outcomes included anxiety [State-Trait Anxiety Inventory (STAI), Visual Analog Scale (VAS), or Child Anxiety Metacognition Scale (CAM-S)], heart rate (HR), and peripheral oxygen saturation (SpO_2_). Standardized mean differences (SMDs) or weighted mean differences (WMDs) with 95% confidence intervals (CIs) were pooled using random-effects models. Subgroup analyses were performed by measurement tool, imaging modality, and patient population.

**Results:**

Six RCTs (n=972 patients) were included. Music significantly reduced post-intervention anxiety compared with control (SMD = -1.94, 95% CI: -3.59 to -0.28, *p=0.02*, I²=98.5%). Analysis of pre-to-post change in anxiety also favored music (SMD = -0.74, 95% CI: -1.22 to -0.26, *p=0.003*, I²=80.5%). Music did not significantly affect HR (WMD = -3.99 bpm, 95% CI: -9.65 to 1.67, *p=0.17*, I²=94.6%) or SpO_2_ (WMD = 0.30%, 95% CI: -0.26 to 0.86, *p=0.30*, I²=74.6%). Subgroup analyses suggested potential effect modification by measurement tool and imaging modality, but heterogeneity remained high. Sensitivity analyses confirmed robustness of anxiety outcomes.

**Conclusion:**

Pooled estimates suggest a reduction in anxiety favoring music, but the extreme heterogeneity (I² = 98.5%) indicates that the studies do not share a common effect size; therefore, the pooled estimate may not represent a clinically meaningful common effect. Music appears safe and low-cost, but conclusions should be drawn cautiously. Future high-quality RCTs with standardized protocols are needed to optimize implementation strategies.

**Systematic review registration:**

https://www.crd.york.ac.uk/PROSPERO, identifier CRD420261356881.

## 1 Introduction

Computed tomography (CT) and magnetic resonance imaging (MRI) are cornerstone diagnostic modalities in modern medicine (1, 2). However, these procedures frequently induce moderate to severe anxiety in patients (3, 4). The confined and noisy environment of MRI scanners, the fear of potential findings, and the need for intravenous contrast injection contribute to claustrophobia and heightened stress responses (5, 6). Anxiety not only impairs patient experience but also degrades image quality due to motion artifacts and may necessitate sedation or scan repetition, thereby increasing healthcare costs and reducing throughput (7–9). Pediatric populations are particularly vulnerable, as their inability to remain still often requires general anesthesia, which carries additional risks (10, 11).

Various interventions have been developed to mitigate procedural anxiety. Pharmacological agents such as benzodiazepines are effective but can cause sedation, respiratory depression, and delayed recovery, and they require medical supervision (12, 13). Consequently, non-pharmacological strategies have gained attention. Among these, music intervention—listening to recorded music or receiving live music therapy—has been proposed as a low-cost, easily implementable, and side-effect-free alternative (14–16). Prior systematic reviews have suggested the anxiolytic effect of music in various medical settings, including surgery and intensive care (17–19). However, evidence specifically for CT and MRI remains scattered and inconclusive. Existing individual studies vary considerably in patient populations (adults vs. children), music types (classical, lullabies, patient-preferred songs), control conditions (silence, headphones only, routine care), and outcome measures. A quantitative synthesis is therefore warranted to clarify the overall effect magnitude and to explore sources of heterogeneity.

This meta-analysis aims to systematically evaluate the efficacy of music intervention in reducing anxiety among patients undergoing CT or MRI examinations, compared with control conditions. Secondary outcomes include heart rate and oxygen saturation as physiological indicators of stress. By focusing exclusively on randomized controlled trials (RCTs) published in English, this study seeks to provide high-level evidence for clinical practice and to identify gaps for future research. Unlike pharmacological anxiolytics, music intervention requires no prescription, has no known adverse effects, and can be delivered by any trained staff member, making it particularly attractive for resource-limited settings. Therefore, this meta-analysis focuses on quantifying the effect of music on anxiety as the primary outcome, alongside physiological measures (heart rate and oxygen saturation), to provide evidence-based guidance for clinical practice (20).

## 2 Methods

### 2.1 Protocol and registration

This systematic review and meta-analysis was conducted following the Preferred Reporting Items for Systematic Reviews and Meta-Analyses (PRISMA) 2020 statement (21). The protocol was registered in PROSPERO (registration number: CRD420261356881).

### 2.2 Search strategy

A comprehensive search was performed in five electronic databases: PubMed (MEDLINE), EMBASE (Ovid), Cochrane Central Register of Controlled Trials (CENTRAL), Web of Science (Core Collection), and CINAHL (EBSCOhost). The search strategy combined terms for the imaging modality (e.g., “MRI,” “CT,” “Computed tomography”), the intervention (e.g., “music,” “music therapy,” “auditory stimulation”), the outcome (e.g., “anxiety,” “fear,” “claustrophobia”), and the study design (randomized controlled trial). Language was restricted to English. No other filters were applied. The final search was performed on March 1, 2026. In addition to electronic database searching, we manually screened the reference lists of included studies and relevant reviews. We did not search gray literature or trial registries (e.g., ClinicalTrials.gov) because the scope of this review was limited to published peer-reviewed RCTs. The complete search strategy for each database is provided in **Supplementary Table 1**.

### 2.3 Eligibility criteria

Studies were included if they met the following PICOS criteria: (1) Population: patients of any age undergoing CT or MRI examinations; (2) Intervention: music intervention (recorded or live) delivered before or during the scan; (3) Comparator: any control condition including usual care, silence, headphones without music, or routine nursing care; (4) Outcomes: at least one of the following—anxiety measured by validated instruments (e.g., STAI, VAS, CAM-S), heart rate, or peripheral oxygen saturation; and (5) Study design: randomized controlled trials (RCTs). For multi-arm trials that included a music-only arm alongside combination arms, only the music-only arm and its corresponding control arm were extracted. Exclusion criteria were: (1) non-RCT study designs (e.g., quasi-experimental, observational, case series); (2) interventions combining music with other active therapies (e.g., aromatherapy) unless a music-only arm was separately reported; (3) invalid or incomplete data; (4) non-English publications; and (5) duplicate publications.

### 2.4 Study selection and data extraction

Two reviewers independently screened titles and abstracts against the eligibility criteria. Full texts of potentially relevant articles were retrieved and assessed independently by the same two reviewers. Disagreements were resolved by consensus or by consulting a third reviewer. A standardized data extraction form was used to collect: (1) study characteristics (first author, year, country, design); (2) participant characteristics (age group, sample size, imaging type); (3) intervention details (music type, delivery method, timing); (4) comparator description; (5) outcome measures and assessment time points; and (6) main results (mean, standard deviation, and p-values for intervention and control groups). For crossover trials, only pre-crossover data were extracted.

### 2.5 Risk of bias assessment

The risk of bias of individual RCTs was assessed using the Cochrane Risk of Bias tool (RoB 1) as described in the Cochrane Handbook for Systematic Reviews of Interventions (13). Seven domains were evaluated: random sequence generation, allocation concealment, blinding of participants and personnel, blinding of outcome assessment, incomplete outcome data, selective reporting, and other bias. Each domain was rated as ‘low risk,’ ‘unclear risk’ (some concerns), or ‘high risk.’ Two reviewers performed the assessment independently, with disagreements resolved through discussion. For performance bias (blinding of participants and personnel), we judged studies as ‘high risk’ when music intervention made blinding impossible; outcome assessors were blinded in all studies, resulting in ‘low risk’ for detection bias.

### 2.6 Outcome measures

Because different anxiety instruments were used across studies (STAI, VAS, and CAM-S), we calculated the standardized mean difference (SMD) to allow pooling. The SMD expresses the effect size in common standard deviation units, which is appropriate for combining different continuous scales that measure the same construct (anxiety). Secondary outcomes were heart rate (HR, beats per minute) and peripheral oxygen saturation (SpO_2_, percentage). For HR and SpO_2_, weighted mean differences (WMDs) were used because units were consistent across studies. When a study reported multiple anxiety scales, the one most commonly used across studies (STAI or VAS) was selected.

### 2.7 Statistical analysis

All meta-analyses were performed using Stata (version 17.0, StataCorp LP, College Station, TX, USA). Given anticipated clinical and methodological heterogeneity, a random-effects model (DerSimonian–Laird method) was applied for all pooled estimates (22). Heterogeneity was quantified using the I² statistic, with values of 25%, 50%, and 75% representing low, moderate, and high heterogeneity, respectively (23). A chi-squared test p-value <0.10 was considered indicative of significant heterogeneity (24). Sensitivity analyses were conducted by sequentially omitting each study and recalculating the pooled estimate to assess the influence of individual studies. Subgroup analyses were performed according to measurement tool (CAM-S, STAI, VAS), imaging modality (CT, CCTA, MRI), and patient population (children vs. adults). Due to the limited number of included studies (n ≤ 6 for all outcomes), publication bias was assessed using Begg’s and Egger’s tests with caution (25). All statistical tests were two-tailed, and a p-value <0.05 was considered statistically significant.

## 3 Results

### 3.1 Study selection

**Figure 1:**
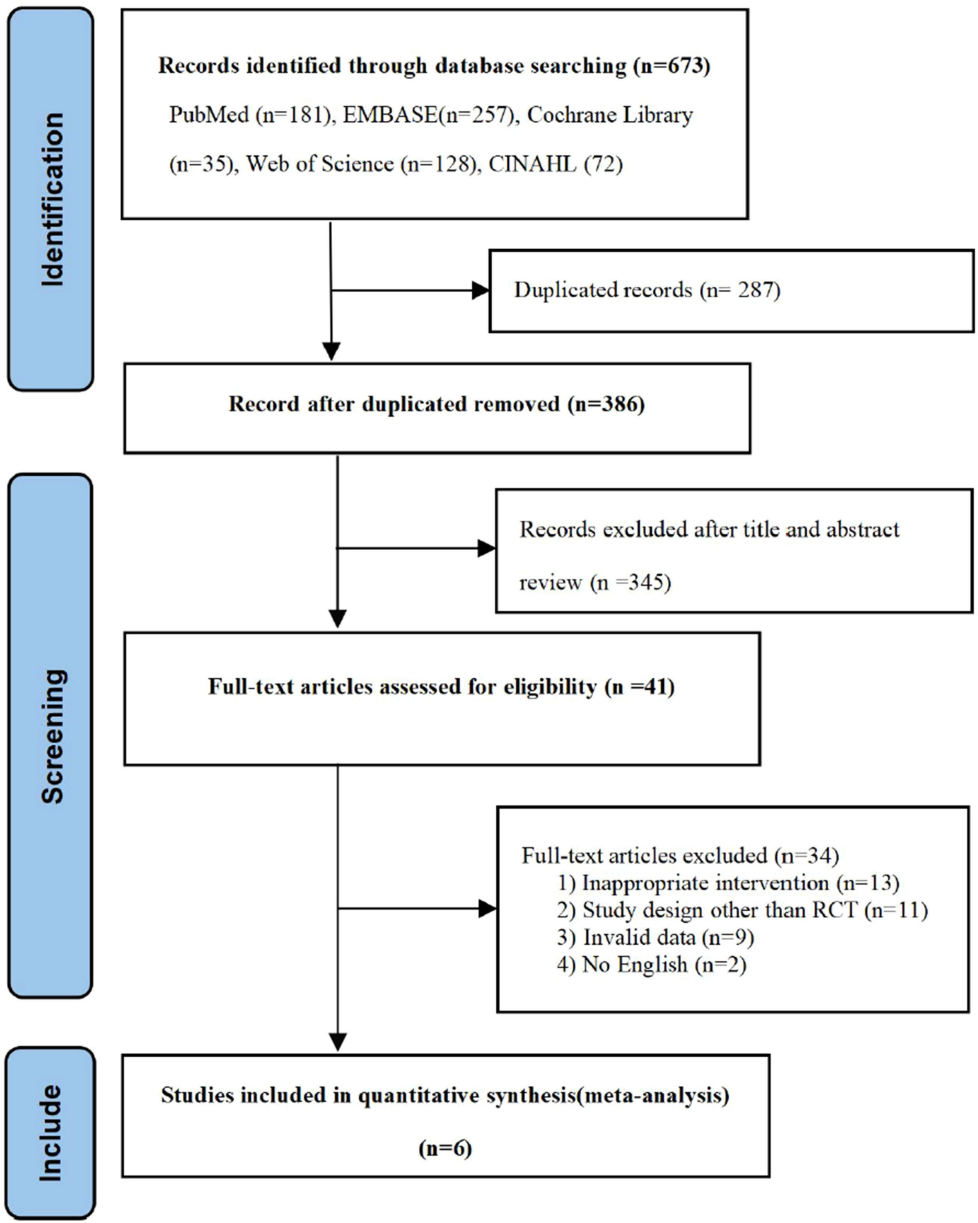
Flowchart of the literature search.

The systematic search yielded 673 records. After removing 287 duplicates, 386 records were screened by title and abstract. Of these, 345 were excluded as irrelevant. The remaining 41 full-text articles were assessed for eligibility, of which 34 were excluded for the following reasons: inappropriate intervention (n=13), study design other than RCT (n=11), invalid data (n=9), and non-English (n=2). Ultimately, 6 RCTs met the inclusion criteria and were included in the quantitative synthesis (26–31). The literature selection process is illustrated in **Figure 1**.

### 3.2 Study characteristics

The six RCTs were published between 2015 and 2025 and included a total of 972 patients (intervention: n=484; control: n=488). Three studies were conducted in Turkey (26, 27, 30), one in the USA (29), one in Canada (28), and one in China (31). The patient populations included children (3–12 years) in two studies (26, 27), infants/toddlers (≤3 years) in one study (29), and adults in the remaining three studies (28, 30, 31). Imaging types were MRI in two studies (26, 31), CT in two studies (27, 29), and coronary CT angiography (CCTA) in two studies (28, 30). Music interventions varied in duration (10–45 min), timing, and delivery. Four studies delivered music only during the scan, one before and during the scan (30) (30–45 min waiting room), and one only before the scan (29). Headphones were MRI-compatible noise-canceling (26, 31), regular padded (27, 28), or none (29) (ambient music). Music was researcher-selected in five studies (classical, lullabies, instrumental) and patient-preferred in one (31). Volume (50–60 dB) was reported in three studies; no adverse effects occurred. Timing of anxiety assessment also varied: immediately post-CT (27), post-recovery (28), immediately post-head CT (29), post-CCTA (30), and immediately post-MRI (31). No study assessed anxiety during the scan. Control conditions included headphones only (no music) (26, 27), routine care without music (29, 31), and standard waiting room (30) or no music (28). The primary outcome anxiety was measured using STAI, VAS, or CAM-S. For Wen et al. (31), which had a four-arm design (aromatherapy+music, aromatherapy only, music only, and routine care), only the music-only arm and the routine care arm were extracted, in accordance with our exclusion criterion that interventions combining music with other active therapies were excluded unless a music-only arm was separately reported. Baseline characteristics and main results are summarized in **Table 1**.

**Table 1:** Baseline characteristics and main results of included trials.

Study	Country	Design	Population	Sample (I/C)	Intervention	Comparator	Outcomes
Gergin et al., 2023 (26)	Turkey	3-arm RCT	Pediatrics (3-12y), MRI	30/30/30 (I1: music, I2: mother voice)	I1: Vivaldi (headphones); I2: mother’s voice (headphones)	Headphones only (no sound)	HR; SpO_2_
Kurudirek et al., 2025 (27)	Turkey	RCT, double-blind	Pediatrics (6-10y), CT	30/30	Mozart lullaby (headphones)	Headphones only (no music)	Anxiety (CAM-S); HR; SpO_2_
Ng et al., 2016 (28)	Canada	RCT	Adults, cardiac CT (CCTA/CCS/PVCT)	100/97	Relaxation music (classical/jazz/new age)	No music	Anxiety (STAI); HR
Park et al., 2015 (29)	USA	RCT, single-blind	Infants/toddlers (≤3y), head CT	30/32	Nursery songs + heartbeat sounds	Usual care (no music)	Anxiety (VAS)
Tekinhatun et al., 2025 (30)	Turkey	Prospective RCT	Adults undergoing CCTA	108/108	Designed waiting room: relaxing music (guitar/piano/flute)	Standard waiting room (no music)	Anxiety (STAI); HR
Wen et al., 2022 (31)	China	RCT, double-blind	Adults (mean 48.3y), MRI	50/50/50/50 (AMTG, AG, MG, RG)	AMTG: Aromatherapy plus music intervention; AG: lavender oil; MG: music only	RG: routine care (video + breathing relaxation)	Anxiety (STAI-1)

AG, Aromatherapy Group; AMTG, Aromatherapy plus Music Therapy Group; C, Control Group; CAM-S, Child Anxiety Metacognition Scale (27); CCTA, Coronary Computed Tomography Angiography; CT, Computed Tomography; HR, Heart Rate; I, Intervention Group; MG, Music Therapy Group; MRI, Magnetic Resonance Imaging; NS, Not Significant; PVCT, Pulmonary Vein CT (28); RCT, Randomized Controlled Trial; RG, Routine Care Group (31); SpO_2_, Peripheral Oxygen Saturation; STAI, State-Trait Anxiety Inventory; VAS, Visual Analog Scale.

### 3.3 Risk of bias

**Figure 2:**
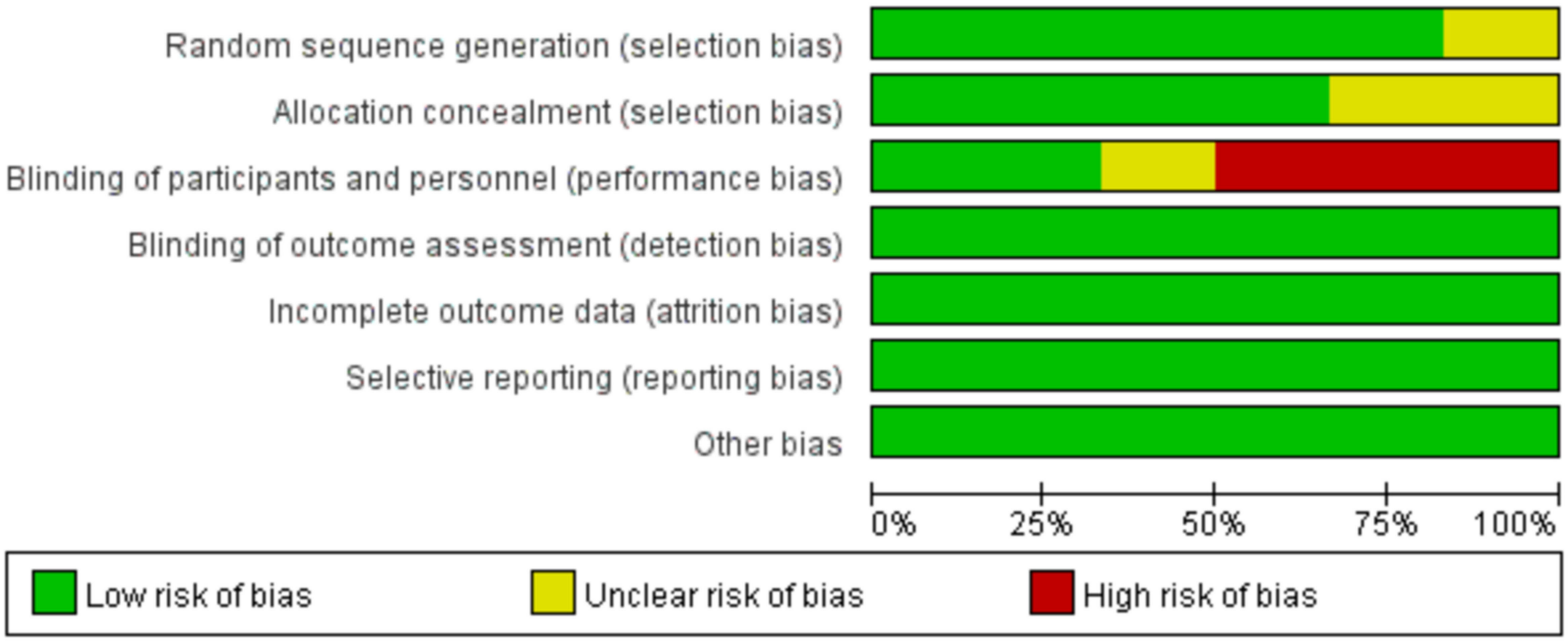
Risk of bias graph.

**Figure 3:**
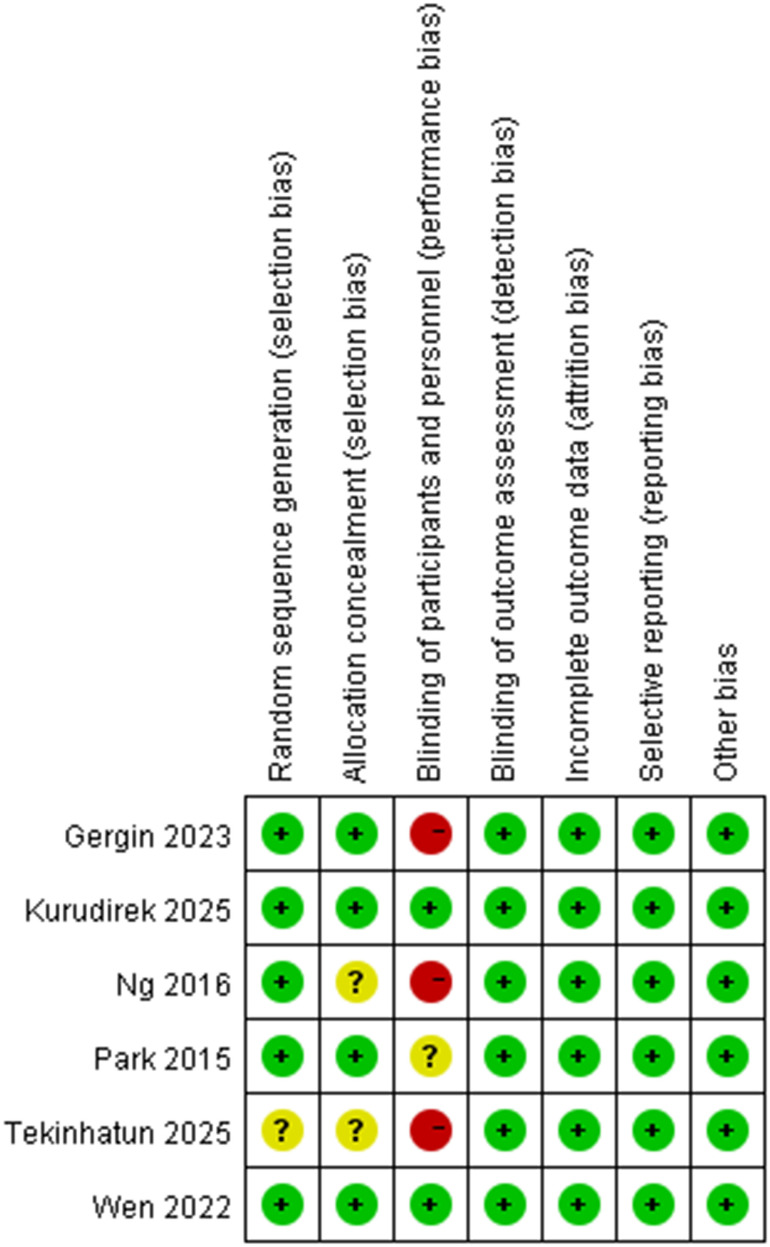
Risk of bias summary.

The risk of bias assessment is presented in **Figures 2** and **3**. Overall, most studies showed low risk of bias for random sequence generation (83.3%) and allocation concealment (66.6%). All studies had low risk of bias for blinding of outcome assessors, incomplete outcome data, and selective reporting. Performance bias (blinding of participants and personnel) was high in 50% of studies because the music intervention inevitably unblinded participants. One study (30) had unclear risk for random sequence generation, two studies (28, 30) for allocation concealment, and one study (29) for blinding of participants and personnel due to partial blinding (nurses were aware of group assignment).

### 3.4 Effect of music on anxiety

#### 3.4.1 Post-intervention anxiety

**Figure 4:**
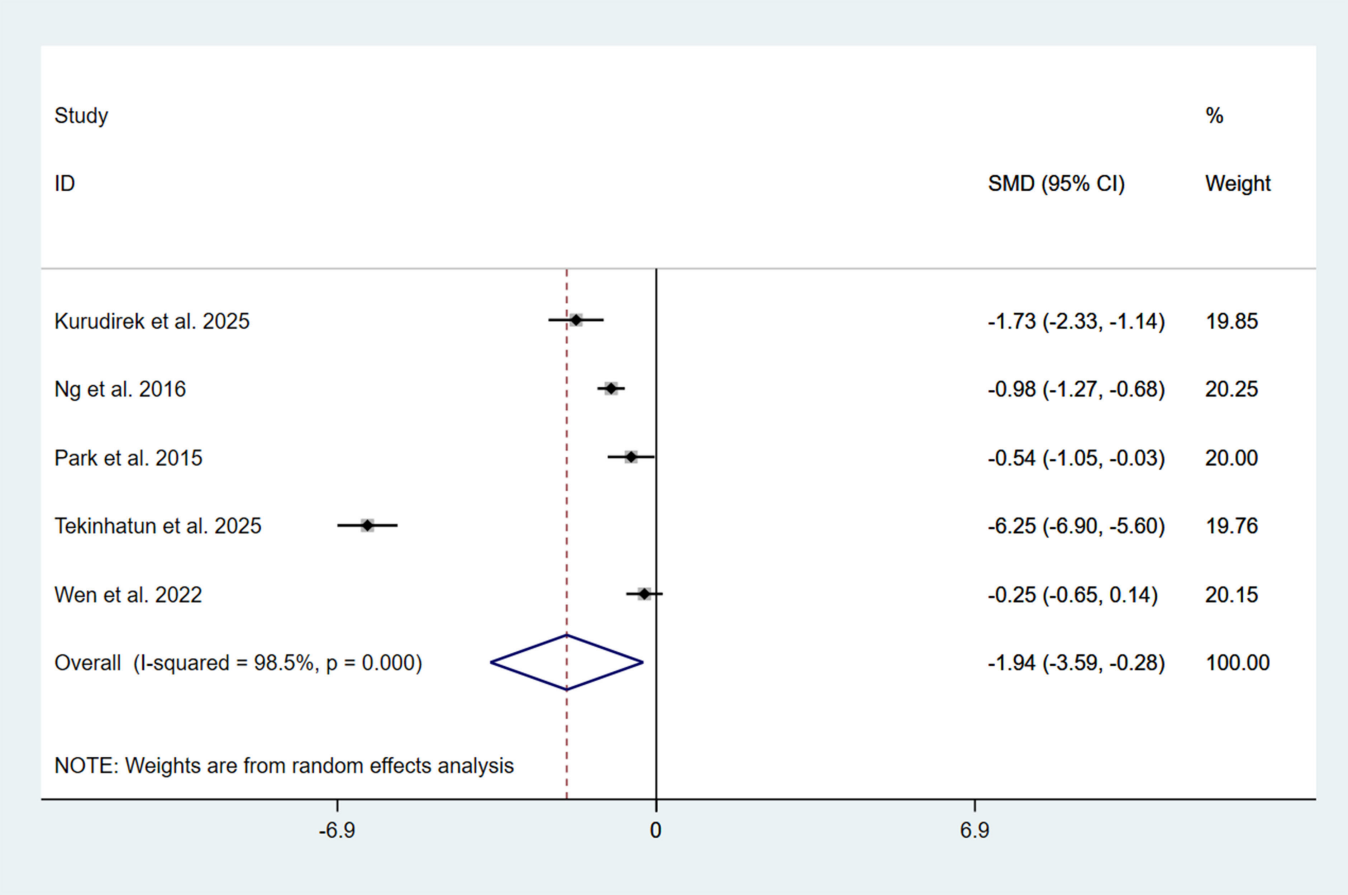
Forest plot of the effect of music intervention on anxiety compared with control in patients undergoing CT or MRI examinations.

Five studies (n=826) reported post-intervention anxiety scores that could be pooled (27–31). The meta-analysis revealed a statistical reduction in anxiety that favored music (SMD = -1.94, 95% CI: -3.59 to -0.28, *p=0.02*), but the very high heterogeneity (I² = 98.5%) limits the interpretability of this pooled estimate as a common effect. The forest plot is shown in **Figure 4**. Tekinhatun et al. (30) reported an exceptionally large effect (SMD = -6.25), which contributed to the heterogeneity.

#### 3.4.2 Change in anxiety (pre-to-post)

**Figure 5:**
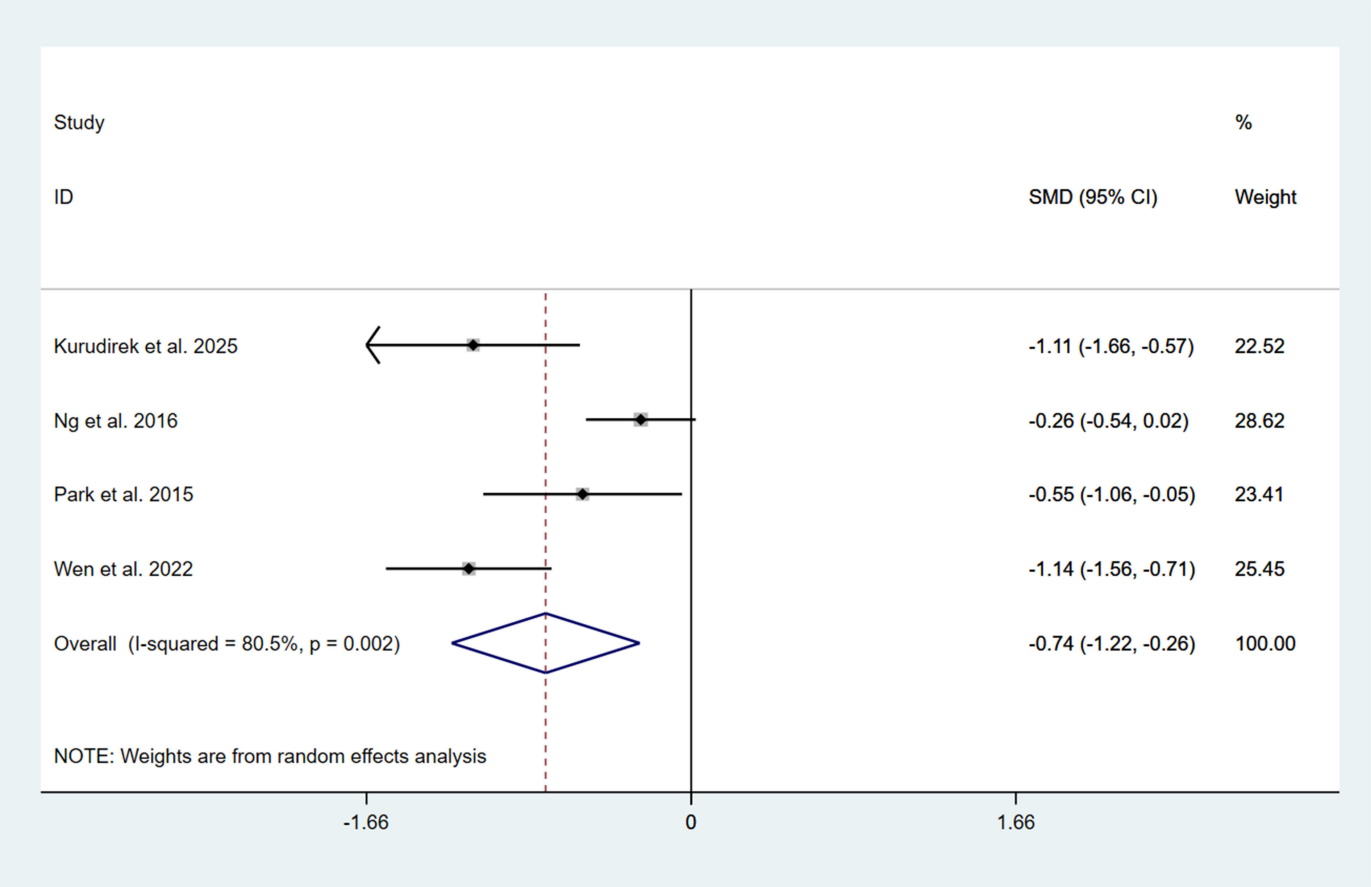
Forest plot of the change in anxiety from pre− to post−intervention (music vs. control) in patients undergoing CT or MRI examinations.

Four studies (n=717) reported pre-to-post intervention changes in anxiety (27–29, 31). The pooled SMD also favored music (SMD = -0.74, 95% CI: -1.22 to -0.26, *p=0.003*), with substantial heterogeneity (I² = 80.5%, *p=0.002*). **Figure 5** presents the forest plot. Kurudirek et al. (27) and Wen et al. (31) showed strong effects (SMD = -1.11 and -1.14, respectively), whereas Ng et al. (28) showed a near-null effect.

#### 3.4.3 Subgroup analyses

Subgroup analyses for post-intervention anxiety (**Table 2**) showed that studies using the STAI (3 studies) had a pooled SMD of -2.48 (95% CI: -5.20 to 0.25) with 99.2% heterogeneity, while the single study using CAM-S (Kurudirek et al. (27)) yielded an SMD of -1.73 (95% CI: -2.33 to -1.14), and the single study using VAS (Park et al. (15)) yielded an SMD of -0.54 (95% CI: -1.05 to -0.03). By imaging modality, CT studies (2 studies) gave an SMD of -1.13 (95% CI: -2.29 to 0.04, I²=88.7%), CCTA studies (2 studies) gave an SMD of -3.60 (95% CI: -8.77 to 1.56, I²=99.5%), and the single MRI study (Wen et al. (31)) gave an SMD of -0.25 (95% CI: -0.65 to 0.14). By patient population, children (2 studies) had an SMD of -1.13 (95% CI: -2.29 to 0.04, I²=88.7%) and adults (3 studies) had an SMD of -2.48 (95% CI: -5.20 to 0.25, I²=99.2%).

**Table 2:** Subgroup analysis for post intervention anxiety (music vs. control).

Subgroup variable	Subgroup	No. of studies	SMD (95% CI)	I² (%)	p for heterogeneity
Measurement tool	CAM-S	1	-1.73 (-2.33, -1.14)	–	–
	STAI	3	-2.48 (-5.20, 0.25)	99.2	<0.001
	VAS	1	-0.54 (-1.05, -0.03)	–	–
Patient population	Children	2	-1.13 (-2.29, 0.04)	88.7	0.003
	Adults	3	-2.48 (-5.20, 0.25)	99.2	<0.001
Imaging modality	CT	2	-1.13 (-2.29, 0.04)	88.7	0.003
	CCTA	2	-3.60 (-8.77, 1.56)	99.5	<0.001
	MRI	1	-0.25 (-0.65, 0.14)	–	–

SMD, standardized mean difference; CI, confidence interval; CAM-S, Child Anxiety Metacognition Scale; STAI, State-Trait Anxiety Inventory; VAS, Visual Analog Scale; CCTA, Coronary Computed Tomography Angiography. A single study does not allow calculation of I² or p for heterogeneity.

For pre-to-post change in anxiety (**Table 3**), subgroup analyses by measurement tool showed that the single CAM-S study had an SMD of -1.11 (95% CI: -1.66 to -0.57), STAI studies (2 studies) had an SMD of -0.68 (95% CI: -1.54 to 0.18, I²=91.3%), and the single VAS study had an SMD of -0.55 (95% CI: -1.06 to -0.05). By imaging modality, CT studies (2 studies) gave an SMD of -0.82 (95% CI: -1.37 to -0.28, I²=53.8%), the single CCTA study (Ng et al. (28)) gave an SMD of -0.26 (95% CI: -0.54 to 0.02), and the single MRI study (Wen et al. (31)) gave an SMD of -1.14 (95% CI: -1.56 to -0.71). By patient population, children (2 studies) had an SMD of -0.82 (95% CI: -1.37 to -0.28, I²=53.8%) and adults (2 studies) had an SMD of -0.68 (95% CI: -1.54 to 0.18, I²=91.3%).

**Table 3:** Subgroup analysis for pre-to-post change in anxiety (music vs. control).

Subgroup variable	Subgroup	No. of studies	SMD (95% CI)	I² (%)	p for heterogeneity
Measurement tool	CAM-S	1	-1.11 (-1.66, -0.57)	–	–
	STAI	2	-0.68 (-1.54, 0.18)	91.3	0.001
	VAS	1	-0.55 (-1.06, -0.05)	–	–
Imaging modality	CT	2	-0.82 (-1.37, -0.28)	53.8	0.141
	CCTA	1	-0.26 (-0.54, 0.02)	–	–
	MRI	1	-1.14 (-1.56, -0.71)	–	–
Patient population	Children	2	-0.82 (-1.37, -0.28)	53.8	0.141
	Adults	2	-0.68 (-1.54, 0.18)	91.3	0.001

SMD, standardized mean difference; CI, confidence interval; CAM-S, Child Anxiety Metacognition Scale; STAI, State-Trait Anxiety Inventory; VAS, Visual Analog Scale; CCTA, Coronary Computed Tomography Angiography. A single study does not allow calculation of I² or p for heterogeneity.

### 3.5 Effect of music on heart rate

**Figure 6:**
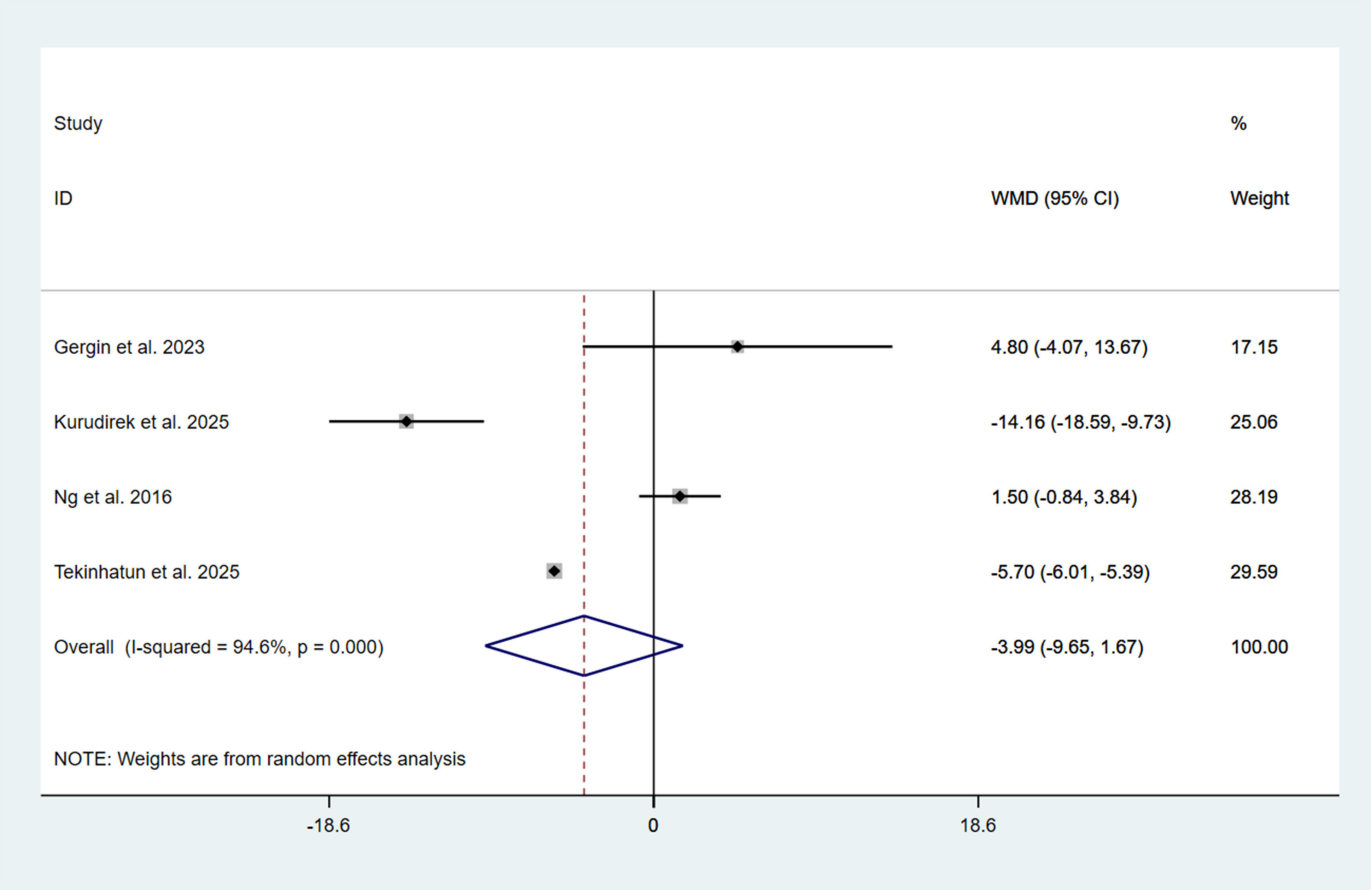
Forest plot of the effect of music intervention on HR compared with control in patients undergoing CT or MRI examinations.

Four studies (n=750) reported HR outcomes (26–28, 30). The pooled WMD was -3.99 bpm (95% CI: -9.65 to 1.67, *p=0.17*), indicating no statistically significant effect. Heterogeneity was high (I² = 94.6%, *p<0.001*). As shown in **Figure 6**, Kurudirek et al. (27) reported a significant reduction (WMD = -14.16 bpm), while Ng et al. (28) and Gergin et al. (26) found no effect.

### 3.6 Effect of music on oxygen saturation

**Figure 7:**
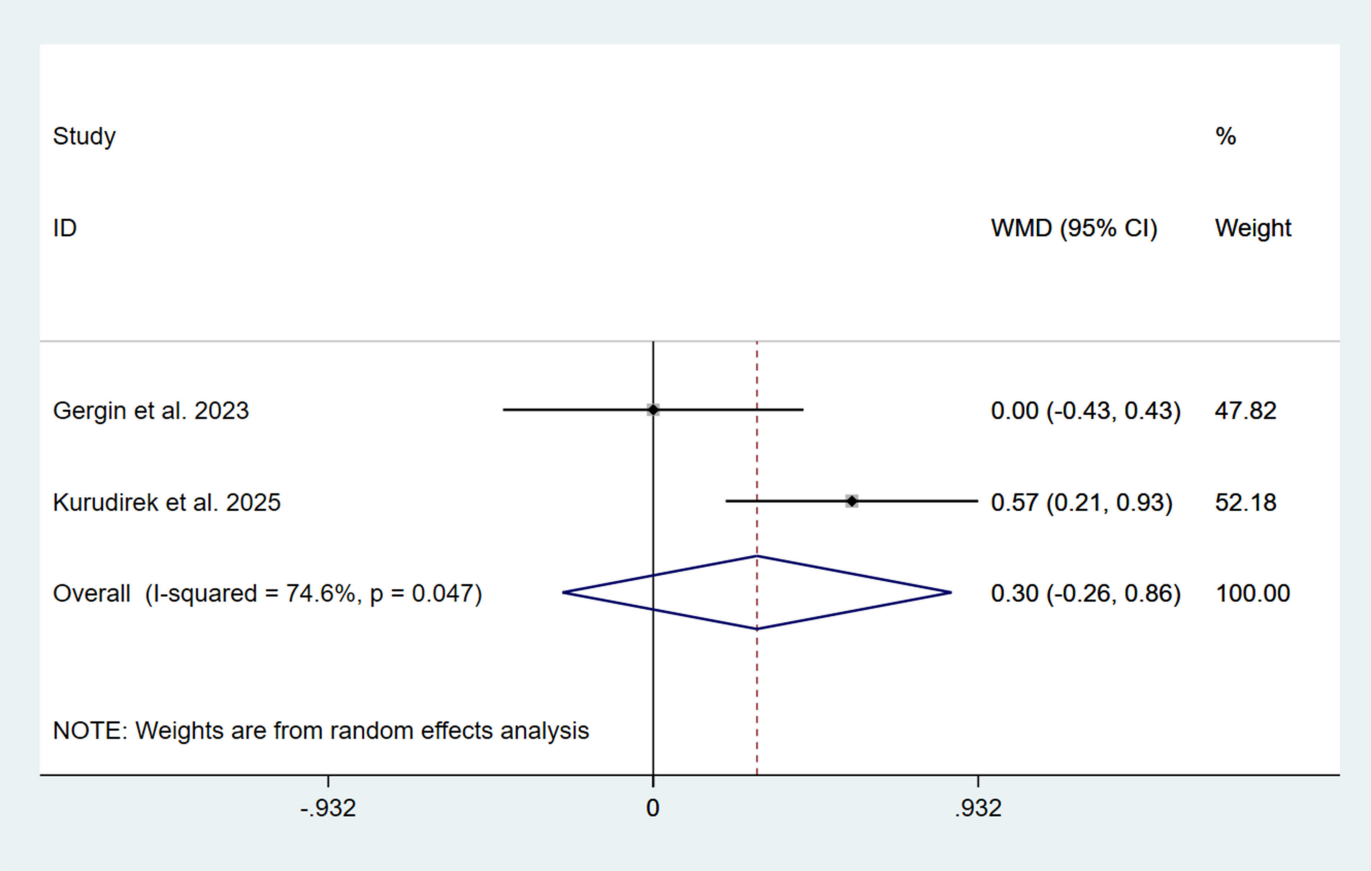
Forest plot of the effect of music intervention on SpO2 compared with control in patients undergoing CT or MRI examinations.

Two studies (n=180) reported SpO_2_ outcomes (26, 27). The pooled WMD was 0.30% (95% CI: -0.26 to 0.86, *p=0.30*), with no significant effect and moderate heterogeneity (I² = 74.6%, *p=0.047*) (**Figure 7**).

### 3.7 Sensitivity analyses

**Figure 8:**
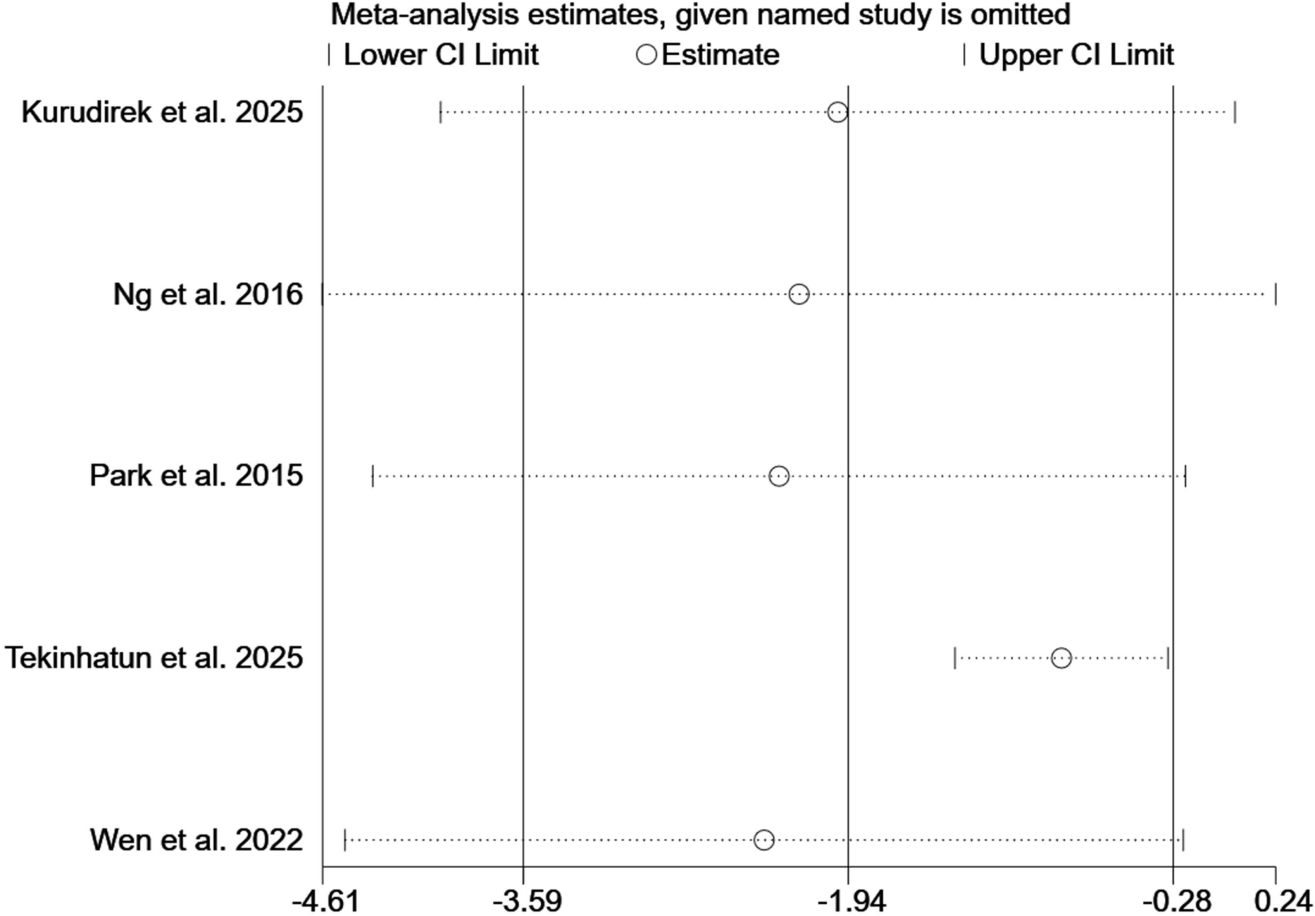
Sensitivity analysis for the effect of music intervention on anxiety in patients undergoing CT or MRI examinations.

**Figure 9:**
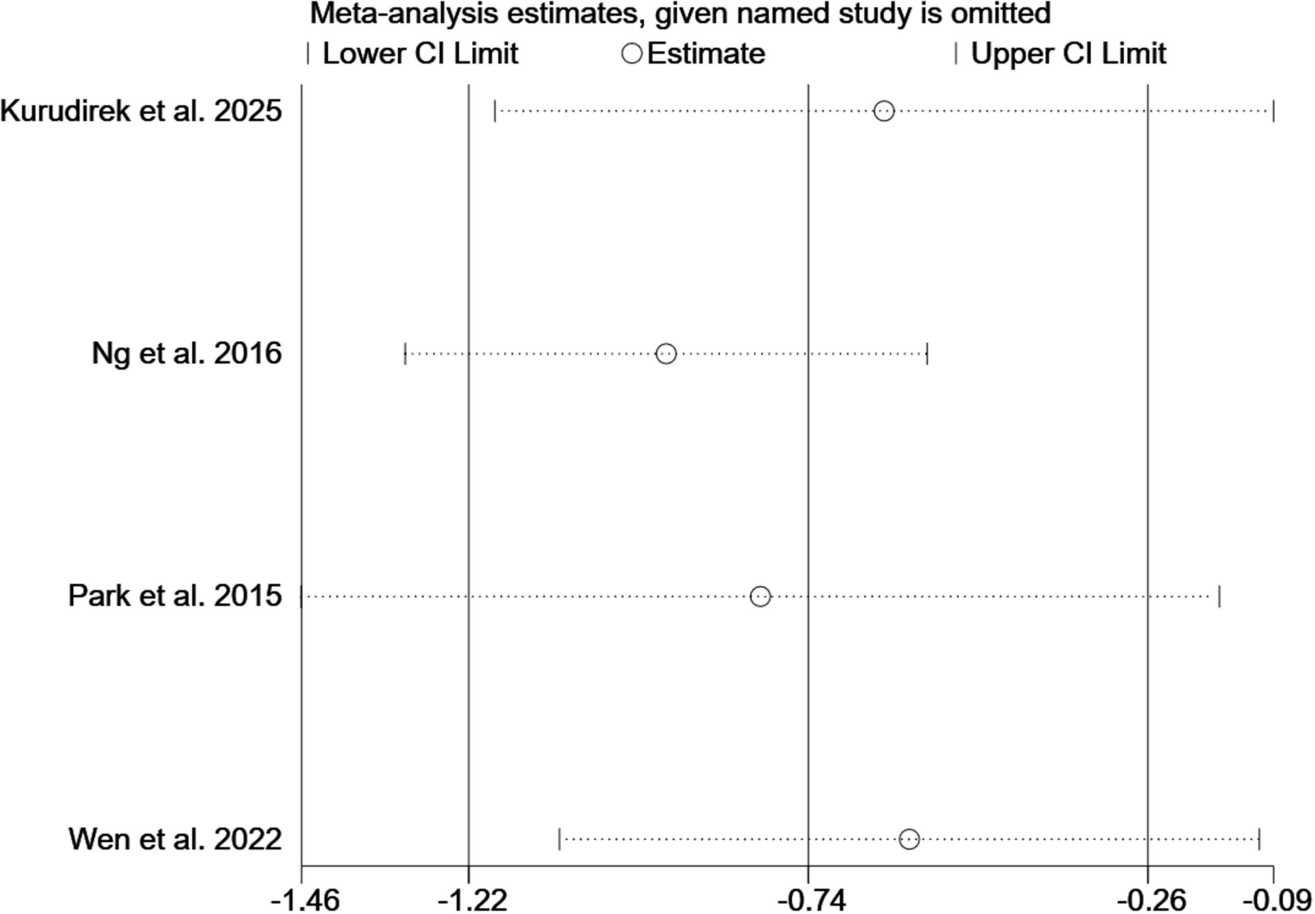
Sensitivity analysis for the change in anxiety from pre− to post−intervention (music vs. control) in patients undergoing CT or MRI examinations.

Sensitivity analyses for the primary outcome (post-intervention anxiety) showed that excluding any single study did not materially change the pooled estimate or its confidence interval, as the lower and upper limits remained at -3.59 and -1.94, respectively (**Figure 8**). For the pre-to-post change in anxiety, sequential omission of each study did not shift the confidence interval to include the null (**Figure 9**), confirming robustness.

### 3.8 Publication bias assessment

Publication bias was assessed for the primary outcome (post-intervention anxiety, 5 studies). For post-intervention anxiety, Begg’s test showed no evidence of asymmetry (adj. Kendall’s score = -7, z = -1.32, *p = 0.188*) and Egger’s test also did not indicate significant small-study effects (bias coefficient = -14.85, 95% CI: -46.90 to 17.20, *p = 0.268*). However, these tests have low power when fewer than 10 studies are included; therefore, the absence of statistical significance should not be interpreted as evidence of no publication bias. The small number of studies precludes a reliable funnel plot assessment. Publication bias remains possible, as negative or null studies may be unpublished.

## 4 Discussion

This systematic review and meta-analysis of six RCTs involving 972 patients provides evidence that music intervention is associated with a reduction in anxiety, but the extreme heterogeneity (I² = 98.5%) suggests that the studies do not share a common effect, and the pooled estimate should be interpreted as an average of highly variable effects rather than a precise clinical effect. The pooled effect size for post-intervention anxiety (SMD = -1.94) would conventionally be considered large according to Cohen’s criteria; however, given the extreme heterogeneity, this estimate may not represent a consistent or clinically meaningful common effect across different settings. The analysis of pre-to-post change in anxiety yielded a more conservative but still significant pooled SMD of -0.74, corresponding to a moderate effect. Importantly, music did not significantly alter heart rate or oxygen saturation, suggesting that its anxiolytic effect may operate through psychological rather than profound physiological pathways—or that physiological responses are more variable and context-dependent.

These findings align with previous meta-analyses of music in other medical contexts. For instance, one meta-analysis demonstrated that listening to music significantly improved preoperative anxiety in patients undergoing gynecological surgery (WMD = -6.78, 95% CI: -7.54 to -6.03) (32). Another meta-analysis reported that perioperative music interventions significantly decreased anxiety compared with controls (MD = -0.69, 95% CI: -0.88 to -0.50) (33). The larger effect observed in our study may be attributable to the inherently anxiety-provoking nature of radiological procedures, in which patients remain awake, alone, and exposed to loud noises or confined spaces. Alternatively, it could reflect publication bias or the inclusion of small studies with exaggerated effects.

It is noteworthy that the effect on anxiety was not paralleled by significant changes in HR or SpO_2_. This dissociation is consistent with the notion that anxiety is a multidimensional construct encompassing cognitive, affective, and physiological components (34, 35). Music may primarily reduce the subjective experience of anxiety (i.e., worry and fear) without consistently altering autonomic arousal, especially in non-stressful conditions (36). Alternatively, the timing of HR measurement (often immediately before or after the scan) may not capture the peak physiological response. Future studies should consider continuous HR monitoring and use of additional biomarkers such as salivary cortisol or skin conductance.

The anxiolytic effect of music can be understood through several complementary mechanisms, drawing from neuroscience, psychology, and psychoneuroimmunology. First, attention diversion is the most parsimonious explanation. Music competes for limited attentional resources, thereby reducing the patient’s focus on threatening stimuli such as scanner noise, claustrophobic sensations, or anticipation of contrast injection (37). This model is supported by studies showing that patient-preferred music is more effective than standardized music, as personal relevance increases attentional engagement (38). The absence of a significant HR reduction in our meta-analysis does not contradict this mechanism, because cognitive reappraisal does not always translate into autonomic changes. Second, mood regulation and emotional contagion play a role. Music with slow tempo, predictable structure, and consonant harmonies activates the parasympathetic nervous system via the nucleus accumbens and ventral tegmental area, leading to dopamine release and reduced cortisol secretion (39, 40). In our included studies, relaxing classical music (e.g., Mozart lullabies) was effective, whereas upbeat or unfamiliar music might have opposite effects (41). However, none of the included studies systematically measured cortisol, leaving this pathway inferential. Third, expectancy and placebo effects cannot be discounted. Patients who receive music may believe that staff are caring for their comfort, thereby reducing anxiety regardless of the auditory stimulus itself. This is why control conditions such as “headphones only without music” are superior to “no headphones,” as they control for the tactile and visual components of the intervention. The study by Gergin et al. (26) explicitly compared mother’s voice versus music, finding both superior to silence, suggesting that any familiar or pleasant auditory stimulus may be beneficial. Fourth, masking of aversive auditory stimuli is particularly relevant for MRI, where the scanner produces noise levels of up to 110 dB (42). Music headphones provide simultaneous sound masking, reducing the startle response and perceived loudness (43). This mechanism is specific to MRI and may explain why the effect sizes for MRI studies (e.g., Wen et al. (31), SMD = -0.25) were not uniformly larger than those for CT studies, as CT is relatively quiet. Notably, the mechanism may differ across age groups. In pediatric populations, music may also serve as a distraction-based coping strategy that empowers children with a sense of control (44, 45). Kurudirek et al. (27) used a Mozart lullaby and reported the largest effect on anxiety among pediatric studies, possibly because lullabies are inherently soothing and associated with parental care.

The findings of this meta-analysis have direct implications for radiology practice. First, music intervention is a low-cost, scalable, and side-effect-free anxiolytic. Unlike benzodiazepines, which require prescriptions, monitoring for respiratory depression, and post-procedure recovery time, music can be delivered by any radiology technician or nurse without additional training (20, 46). The cost of purchasing headphones and a music player is negligible compared with the cost of a single sedated scan or repeated scans due to motion artifacts. Radiology departments may consider offering music as a complementary comfort measure, particularly for patients with known claustrophobia, children, and those undergoing lengthy procedures, while acknowledging that the evidence is based on a small number of heterogeneous studies. Second, the choice of music matters. Although our meta-analysis could not compare different music types directly due to heterogeneity, the available evidence suggests that patient-preferred music (31), lullabies for children (27), and relaxing instrumental music (28, 30) are effective. A practical approach would be to offer a menu of recorded options (classical, nature sounds, ambient, or patient’s own playlist) via a hospital-provided tablet or personal smartphone. Third, the timing and duration of music exposure require optimization. In the included studies, music was delivered from 10 minutes before to throughout the scan. However, no study compared different durations. It is plausible that the effect builds over time, with a minimum effective duration of 15 minutes, as seen in surgical settings (47). Departments could start music in the waiting room (as in Tekinhatun et al. (30)) and continue during the scan to maintain the effect.

For policy makers, this evidence suggests that music intervention could be considered in future discussions regarding patient-centered care in radiology; however, the limited number of studies and high heterogeneity preclude strong recommendations for inclusion in national guidelines at this time. The Joint Commission and the American College of Radiology have endorsed non-pharmacological comfort measures, but specific recommendations on music are lacking (48). Given the patient preference for music and the absence of harms, music could be explored as a potential quality indicator for patient-centred care, but further evidence is needed to standardize its implementation before formal endorsement (20). Moreover, reimbursement models such as value-based purchasing could incentivize radiology departments to implement music as a low-cost intervention that improves patient satisfaction and reduces sedation use.

Based on the findings of this meta-analysis, we outline below a tentative, evidence-informed example of how music might be offered, acknowledging that these suggestions are drawn from a small number of heterogeneous studies and require further validation. Radiology departments that wish to pilot music interventions may consider the following elements: (1) Music selection: Patient-preferred music is recommended when feasible (as in 31); otherwise, relaxing instrumental music with slow tempo (60–80 beats per minute), consonant harmonies, and soft timbre (e.g., piano, flute, classical guitar) should be used. Lullabies are particularly effective for children (27). (2) Timing and duration: Music could start at least 10–15 minutes before the scan (ideally in the waiting room) and continue throughout the examination. A total duration of 30–45 minutes (as in 30) appears to yield larger effects, but even shorter durations (e.g., 10 minutes) are beneficial. (3) Delivery method: For MRI, MRI-compatible noise-canceling headphones are essential; for CT, regular padded headphones or ambient speakers (if noise levels permit) can be used. Volume should be set at a comfortable level (50–60 dB) and never exceed 70 dB to avoid hearing discomfort. (4) Staff training: No specialized training is required; a radiology technician or nurse can initiate the music. A simple checklist (headphones checked, music started, volume confirmed) ensures standardization. (5) Documentation: Recording whether music was offered and the patient’s acceptance may be helpful for local quality improvement. We emphasize that this is not a validated clinical guideline but a synthesis of available, albeit heterogeneous, study protocols. Future research should test such protocols in well-designed, adequately powered RCTs before widespread adoption.

The very high heterogeneity (I² > 80% for all outcomes) is a major concern. Several sources likely contributed to the extreme heterogeneity. First, clinical diversity was substantial: the two pediatric studies used the CAM-S scale and reported SMDs of -1.73 and -1.11, whereas adult studies used STAI or VAS and produced a wider range of effects (SMD from -0.25 to -6.25). Nevertheless, the use of different anxiety scales (STAI, VAS, CAM-S) may have contributed to heterogeneity. Although the SMD was designed to combine such diverse instruments, the scales differ in their sensitivity to acute procedural anxiety (e.g., VAS is more responsive to moment-to-moment changes, while STAI measures longer-lasting states). The extreme effect from Tekinhatun et al. (STAI) and the moderate effect from Park et al. (VAS) illustrate this variability. Readers should therefore interpret the pooled SMD as a general tendency rather than a precisely calibrated effect. Second, imaging modality appeared to modify the effect: MRI studies showed a non-significant SMD of -0.25, CT studies a moderate SMD of -1.13, and CCTA studies a very large but imprecise SMD of -3.60. The different acoustic environments (MRI scanner noise vs. quiet CT) likely influence the masking and distraction mechanisms of music. Third, music type and duration varied considerably: Tekinhatun et al. used 30–45 minutes of relaxing instrumental music in the waiting room, yielding an extreme effect (SMD = -6.25), whereas Wen et al. used patient-preferred music only during the scan with a null effect. Fourth, the timing of outcome assessment differed: most studies measured anxiety immediately after the scan, but some measured after transfer to recovery or even before scan completion. Fifth, control conditions ranged from ‘headphones only’ (which controls for tactile and visual cues) to ‘no music’ or ‘usual care’, with more rigorous controls tending to produce smaller effects. Finally, statistical heterogeneity was inflated by the use of a random-effects model, which assumes that true effects vary across studies. Given this high heterogeneity, the pooled estimate should be interpreted as a general indication of benefit rather than a precise effect size. At I² = 98.5%, many methodologists would question whether a single pooled effect is interpretable at all. Therefore, the summary estimate should be viewed as an average of disparate study results rather than a precise clinical parameter.

Subgroup analyses suggested that measurement tool and imaging modality may explain some but not all heterogeneity, as I² remained high within subgroups (e.g., STAI subgroup I²=99.2%).

This meta-analysis has several limitations. First, the small number of included studies (n=6) limits the precision of pooled estimates and precludes meta-regression or reliable publication bias assessment. Second, language restriction to English may have introduced language bias, as two non-English RCTs were excluded. Third, inability to blind participants and personnel (performance bias) is inherent to music interventions; although outcome assessors were blinded, subjective outcomes like anxiety may be influenced by patient expectation. Fourth, substantial clinical and methodological heterogeneity (I² = 98.5%) across studies – due to differences in age groups (children vs. adults), imaging modalities (CT, CCTA, MRI), music types (lullabies, classical, patient-preferred), intervention duration (10–45 min), timing (before/during scan), and control conditions – reduces confidence in the pooled effect size. Fifth, limited pediatric data: only two studies included children, and one of these focused on infants (≤3 years), so findings may not be generalizable to all pediatric ages. Sixth, absence of long-term outcomes: none of the studies assessed anxiety beyond the immediate post-procedure period, so it is unknown whether the anxiolytic effect persists or affects willingness to return for future scans. Seventh, lack of physiological biomarker measurement (e.g., salivary cortisol, heart rate variability) limits understanding of the mechanisms underlying anxiety reduction. Finally, variability in music delivery (headphone type, volume, patient vs. researcher selection) was not standardized, affecting reproducibility. Furthermore, the included studies lacked detailed reporting of musical characteristics (tempo, pitch timbre, instrument type, raga-based structures), limiting our ability to identify the most effective musical features. Future RCTs should address these limitations by using larger samples, standardized protocols, active control groups, and long-term follow-up.

## 5 Conclusion

This meta-analysis of six RCTs suggests that music intervention may reduce anxiety in patients undergoing CT or MRI examinations, with a moderate to large effect size (SMD = -0.74 to -1.94 depending on outcome definition). However, the very high heterogeneity (I² = 98.5%) and the small number of included studies limit the precision and generalizability of these findings. Music did not significantly affect heart rate or oxygen saturation. Given its low cost, safety, and ease of implementation, offering music to radiology patients (particularly those with high baseline anxiety, children, and individuals undergoing MRI) is reasonable as a complementary comfort measure. Nevertheless, clinicians should be aware that the effect may vary considerably across settings. Larger, methodologically rigorous RCTs with standardized protocols and long-term follow-up are needed before strong clinical recommendations can be made.

## Data Availability

The original contributions presented in the study are included in the article/**Supplementary Material**. Further inquiries can be directed to the corresponding authors.

## Supplementary material

Supplementary Table 1 Full electronic search strategy.
