# Supplementary material for: Music intervention for reducing anxiety in patients undergoing CT or MRI examinations: a systematic review and meta-analysis of randomized controlled trials
Source: Front Psychiatry. 2026 Aug 5;17:1864214. doi: 10.3389/fpsyt.2026.1864214 (PMC13485982; doi:10.3389/fpsyt.2026.1864214)
Supplement: Supplementary file 1 [file SupplementaryFile1.docx]

### **1. PubMed (MEDLINE)**

| Item | Details |
| --- | --- |
| ****Platform**** | PubMed (National Library of Medicine) |
| ****Language Restriction**** | English |
| ****Study Type Restriction**** | Randomized Controlled Trial |
| ****Search Strategy**** | ****#1**** "Magnetic Resonance Imaging"[Mesh] ****#2**** "Tomography, X-Ray Computed"[Mesh] ****#3**** MRI[tiab] OR CT[tiab] OR "computed tomography"[tiab] OR "computerized tomography"[tiab] OR "magnetic resonance imaging"[tiab] ****#4**** #1 OR #2 OR #3 ****#5**** "Music"[Mesh] OR "Music Therapy"[Mesh] ****#6**** music[tiab] OR melody[tiab] OR "auditory stimulation"[tiab] ****#7**** #5 OR #6 ****#8**** "Anxiety"[Mesh] OR "Fear"[Mesh] ****#9**** anxiety[tiab] OR fear[tiab] OR stress[tiab] OR claustrophobia[tiab] OR "patient comfort"[tiab] ****#10**** #8 OR #9 ****#11**** "Randomized Controlled Trial"[Publication Type] OR "Controlled Clinical Trial"[Publication Type] OR randomized[tiab] OR placebo[tiab] OR "clinical trial"[tiab] OR RCT[tiab] ****#12**** #4 AND #7 AND #10 AND #11 ****#13**** #12 AND (english[Filter]) |
| ****Number of Results**** | **181** |

### **2. Embase (Ovid)**

| Item | Details |
| --- | --- |
| ****Platform**** | Embase (Ovid) |
| ****Language Restriction**** | English |
| ****Study Type Restriction**** | Randomized Controlled Trial |
| ****Search Strategy**** | ****#1**** 'magnetic resonance imaging'/exp ****#2**** 'computer assisted tomography'/exp ****#3**** MRI:ab,ti OR CT:ab,ti OR 'computed tomography':ab,ti OR 'computerized tomography':ab,ti OR 'magnetic resonance imaging':ab,ti ****#4**** #1 OR #2 OR #3 ****#5**** 'music'/exp OR 'music therapy'/exp ****#6**** music:ab,ti OR melody:ab,ti OR 'auditory stimulation':ab,ti ****#7**** #5 OR #6 ****#8**** 'anxiety'/exp OR 'fear'/exp ****#9**** anxiety:ab,ti OR fear:ab,ti OR stress:ab,ti OR claustrophobia:ab,ti OR 'patient comfort':ab,ti ****#10**** #8 OR #9 ****#11**** 'randomized controlled trial'/exp OR 'controlled clinical trial':ab,ti OR randomized:ab,ti OR placebo:ab,ti OR 'clinical trial':ab,ti OR rct:ab,ti ****#12**** #4 AND #7 AND #10 AND #11 ****#13**** #12 AND [english]/lim |
| ****Number of Results**** | **257** |

### **3. Cochrane Central Register of Controlled Trials (CENTRAL)**

| Item | Details |
| --- | --- |
| ****Platform**** | Cochrane Library (Wiley) |
| ****Language Restriction**** | English |
| ****Study Type Restriction**** | Randomized Controlled Trial (inherent to CENTRAL) |
| ****Search Strategy**** | ****#1**** (MRI OR CT OR "computed tomography" OR "computerized tomography" OR "magnetic resonance imaging") ****#2**** (music OR melody OR "auditory stimulation") ****#3**** (anxiety OR fear OR stress OR claustrophobia OR "patient comfort") ****#4**** (randomized OR placebo OR "clinical trial" OR RCT) ****#5**** #1 AND #2 AND #3 AND #4 |
| ****Number of Results**** | **35** |

### **4. Web of Science (Core Collection)**

| Item | Details |
| --- | --- |
| ****Platform**** | Web of Science (Clarivate) |
| ****Language Restriction**** | English |
| ****Study Type Restriction**** | Randomized Controlled Trial |
| ****Search Strategy**** | ****#1**** TS=("magnetic resonance imaging" OR "MRI" OR "computed tomography" OR "CT" OR "computerized tomography") ****#2**** TS=(music OR melody OR "auditory stimulation") ****#3**** TS=(anxiety OR fear OR stress OR claustrophobia OR "patient comfort") ****#4**** TS=(randomized OR "randomized controlled trial" OR placebo OR "clinical trial" OR RCT) ****#5**** #1 AND #2 AND #3 AND #4 ****#6**** #5 AND LA=(english) |
| ****Number of Results**** | **128** |

### **5. CINAHL (EBSCOhost)**

| Item | Details |
| --- | --- |
| ****Platform**** | CINAHL (EBSCOhost) |
| ****Language Restriction**** | English |
| ****Study Type Restriction**** | Randomized Controlled Trial |
| ****Search Strategy**** | ****S1**** MH "Magnetic Resonance Imaging+" ****S2**** MH "Tomography, X-Ray Computed+" ****S3**** TI (MRI OR CT OR "computed tomography" OR "computerized tomography" OR "magnetic resonance imaging") OR AB (MRI OR CT OR "computed tomography" OR "computerized tomography" OR "magnetic resonance imaging") ****S4**** S1 OR S2 OR S3 ****S5**** MH "Music+" OR MH "Music Therapy+" ****S6**** TI (music OR melody OR "auditory stimulation") OR AB (music OR melody OR "auditory stimulation") ****S7**** S5 OR S6 ****S8**** MH "Anxiety+" OR MH "Fear+" ****S9**** TI (anxiety OR fear OR stress OR claustrophobia OR "patient comfort") OR AB (anxiety OR fear OR stress OR claustrophobia OR "patient comfort") ****S10**** S8 OR S9 ****S11**** MH "Randomized Controlled Trials+" ****S12**** TI (randomized OR placebo OR "clinical trial" OR RCT) OR AB (randomized OR placebo OR "clinical trial" OR RCT) ****S13**** S11 OR S12 ****S14**** S4 AND S7 AND S10 AND S13 ****S15**** S14 AND (LA "english") |
| ****Number of Results**** | **72** |
